# Effects of Interventions to Improve Help-Seeking Related to Mental Health Among Workers: A Systematic Review

**DOI:** 10.7759/cureus.82935

**Published:** 2025-04-24

**Authors:** Susumu Fukita, Hiromi Kawasaki, Kyosuke Yorozuya

**Affiliations:** 1 Department of Health Promotion, National Institute of Public Health, Wako, JPN; 2 Department of Health Science, Graduate School of Biomedical and Health Sciences, Hiroshima University, Hiroshima, JPN; 3 Faculty of Rehabilitation and Care, Seijoh University, Tokai, JPN

**Keywords:** help-seeking, mental health, randomized controlled trial, systematic review, worker

## Abstract

Burdens caused by mental disorders are most common in the working age. Although mental health-related disorders are typically preventable or treatable, individuals are often unable to seek help. Moreover, there is a lack of systematic reviews that elucidate the effectiveness of interventions to improve help-seeking related to workers' mental health, despite the greater burden of mental health issues during the working age. Thus, the current study attempts to address this research gap by conducting a systematic review. A literature search was conducted on five databases (The Cochrane Library, PubMed, CINAHL via EBSCOhost, PsycINFO via EBSCOhost, and Ichushi-Web), concluding on February 1, 2022. The inclusion criteria were randomized controlled trials, quantitative studies, studies whose outcomes included help-seeking related to mental health for workers, and studies that were published in English or Japanese. The exclusion criteria were studies that lacked pretest data for outcome measurements and whose outcomes targeted help-seeking regarding the mental health of military personnel. The quality of the selected studies was assessed using the Modified Jadad Scale. A meta-analysis was not conducted due to the diverse outcomes and measurements for help-seeking. The review was conducted on 10 reports, with participant numbers ranging from 39 to 1,557. Only two out of seven interventions to measure help-seeking attitudes reported effects at posttest. Although two interventions to measure help-seeking intention produced effects at posttest, no study revealed any effect at follow-up beyond six months. One intervention to measure help-seeking behavior pointed to an effect at posttest, and only one intervention reported effects at the one-year follow-up. Regarding interventions to measure informal help, only one found no effect at the three- and six-month follow-up. This study failed to draw conclusions about the effects of interventions due to a lack of related studies. However, the findings underscore the difficulty in improving help-seeking pertaining to mental health for workers. Thus, further research is required to improve help-seeking tailored to the complex characteristics of workers' conditions.

## Introduction and background

Mental health issues remain a major challenge to public health. Mental disorders were a major cause of disability-adjusted life years in 2019 [1]. In other words, mental disorders pose a significant burden to society.

In 2019, scholars reported that a total of 80.6% of the burden caused by mental disorders occurred during the working age [1]. Other studies highlight the link between poor mental health and workers' productivity loss [2]. Furthermore, productivity loss due to anxiety and depression has resulted in a significant global economic loss of US$ 1 trillion [3].

Mental health disorders are preventable or treatable with interventions. Thus, help-seeking, including the use of mental health services, is crucial. In the mental health context, help-seeking is defined as an "adaptive coping process that is the attempt to obtain external assistance to deal with a mental health concern" [4]. However, a survey of 17 countries found mental health services to be underutilized, with utilization rates of just 1.6% and 17.9% in Nigeria and the United States, respectively, over the preceding 12 months [5]. This suggests that individuals are unable to seek help for mental health issues.

Previous studies report that high levels of mental health literacy predict the use of mental health services among those with mental health problems [6]. In addition, stigma exerts a negative effect on help-seeking [7]. These findings suggest that support for promoting help-seeking regarding mental health is crucial.

Intervention studies have been conducted for various aspects of help-seeking, such as attitude, intention, and behavior. In a previous systematic review of randomized controlled trials (RCTs) that promoted help-seeking for depression, anxiety, and general psychological distress, which was not limited to the participants, five studies that examined help-seeking attitudes at posttest reported specific effects [8]. However, one study that assessed help-seeking intention found no effect at posttest, and only one of the three studies that investigated help-seeking behavior found an effect at posttest [8]. Another systematic review of RCTs or non-RCTs not limited to the participants provided evidence of the effectiveness of formal help-seeking attitude, intention, and behavior regarding mental health in adults [9], with the authors highlighting the need to elucidate the effects of interventions for specific target populations. However, despite the greater burden of mental health during working age, systematic reviews to clarify the effectiveness of interventions that target workers are lacking.

This study recognizes the need to provide evidence-based support by conducting a systematic review that elucidates the effects of interventions that promote help-seeking regarding mental health for workers.

## Review

Methodology

Search Strategy

A literature search was conducted using five databases (The Cochrane Library, PubMed, CINAHL via EBSCOhost, PsycINFO via EBSCOhost, and Ichushi-Web). The search strategy was first developed in PubMed and subsequently converted to fit the other databases (for details, please refer to the Appendices section, which illustrates the search strategies pertaining to the five databases). The search terms were "help-seeking", "mental health", and "worker". Medical subject headings (MeSH) terms related to help-seeking and mental health were searched using titles and abstracts. The term "worker" was searched using full texts to include potential articles that were not identified using titles or abstracts, because the term "worker" could represent participants. The search terms were connected using "and" or "or." Filters were used to restrict the language to English or Japanese (except for The Cochrane Library) and the study design to RCTs. RCTs were used in The Cochrane Library, CINAHL, and PsycINFO. The year of publication was not restricted. The literature search using the abovementioned databases was completed on February 1, 2022. A librarian reviewed the search strategy.

Inclusion and Exclusion Criteria

The inclusion criteria were RCTs, quantitative studies, studies whose outcomes included help-seeking related to mental health for workers, and studies that were published in English or Japanese. The exclusion criteria were studies that did not include pretest data of outcome measurements and studies whose outcomes targeted help-seeking regarding the mental health of military personnel.

Study Selection

Duplicate records were omitted and managed using EndNote (Clarivate, Philadelphia, Pennsylvania). Furthermore, duplicate records were manually identified. Two researchers (SF and KY) independently screened the records based on their titles and abstracts, as well as against the inclusion and exclusion criteria. Subsequently, the reports were also assessed for eligibility independently by the two researchers, considering the full texts and the inclusion and exclusion criteria. Their judgments were periodically checked and discussed until a consensus was reached. The search and selection process followed the PRISMA (Preferred Reporting Items for Systematic Reviews and Meta-Analyses) 2020 flow diagram [10].

Quality Assessment

The quality of each selected study was assessed using the Modified Jadad Scale [11-13], which comprises the following six items [11,12]: (a) Was the study described as randomized?; (b) Was the study described as double-blind?; (c) Was there a description of withdrawals and drop outs?; (d) Was there a clear description of the inclusion and exclusion criteria?; (e) Was the method used to assess adverse effects described?; and (f) Were the methods of statistical analysis described?

For each question, "Yes" is assigned a value of 1, and "No" is assigned a value of 0. For item (a), if the methods of randomization were described and if such methods were appropriate, an additional point is given. Otherwise, a point is deducted. Similarly, for item (b), if the double-blind methods were described and if such methods were appropriate, an additional point is given; otherwise, one point is deducted. The scores range from 0 to 8, with higher scores indicating better quality of each study. Specifically, three points or less indicate low quality, whereas four points and above indicate high quality. Two researchers (SF and KY) conducted quality assessments independently, reviewing the results and holding discussions until a consensus was reached.

Data Extraction

SF and KY also independently conducted data extraction. The extracted data were periodically checked and discussed until a consensus was reached. The extracted items were author, year of publication, country, participant, age and sex, number of participants, contents of intervention, duration and intensity of intervention, outcome measures, assessment times, and results.

Synthesis Methods

Although a meta-analysis was not conducted owing to the diverse outcomes and measurements for help-seeking, we calculated the effect sizes between groups (Cohen's d) to compare the results of the selected studies. For studies in which the effect size between groups could not be calculated and was not presented in the report, the effect size within group (Cohen's d) was calculated. If effect sizes were indicated in reports, those results were adopted. For studies in which the effect size (Cohen's d) could not be calculated, we included the results presented in the reports.

Ethical Approval

Because this study is a systematic review, ethical approval was not necessary.

Results

Study Selection

**Figure 1:**
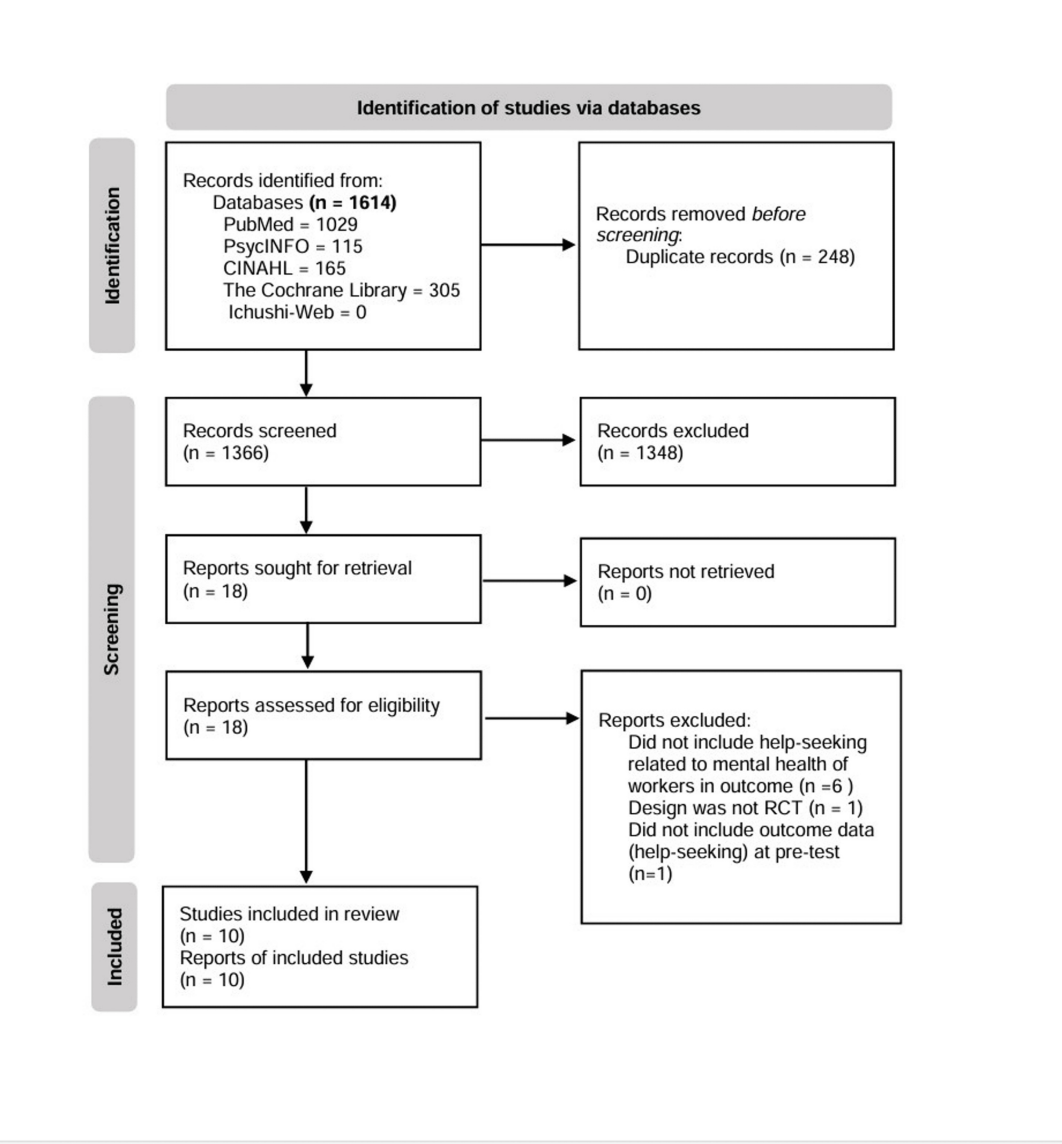
Search and selection process

A total of 1,614 records were extracted from the five databases, and 248 duplicates were excluded. The remaining 1,366 records were screened by their titles and abstracts and the inclusion and exclusion criteria, which resulted in the omission of 1,348 records. Of the remaining 18 reports, which were assessed for eligibility based on the full text and the inclusion and exclusion criteria, eight more reports were excluded. Finally, 10 reports [14-23] were selected for review (Table 1). Figure 1 presents the search and selection process using the PRISMA 2020 flow diagram.

**Table 1:** Characteristics of the selected studies ^a^Employees whose leaders were part of the intervention or control group. AOD: alcohol or drug; CBT: cognitive behavioral therapy; EAPs: employee assistance programs; FU: follow-up; MH-Guru: mental health guru; MHFA: Mental Health First Aid; SOS: signs of struggle scale; SWSW: stay well, stay working

Study	Country	Participants	Age and Sex	Randomized	Post, FU	Groups	Duration	Intensity
Bennett and Lehman (2001) [14]	USA	Municipal employees (excluding uniformed police and fire personnel)	Age between 31 and 40 years (33%) or older (49%). Sex: male (64%)	T = 153, I = 109 C = 118	Post T = 101 I = 73 C = 86 FU T = 64 I = 64 C = 69	T = Team training (embedded messages about AOD reduction in the context of team building and stress management + role-playing) I = Informational training (informational review of EAPs and policy + video + participative quiz + video with follow-up discussion + brief game-oriented quiz) C = Control (no training)	T = 4 hours per session I = 2 hours per session	T and I = two different sessions conducted two weeks apart
Tan et al. (2021) [15]	Australia	Firefighters	Age W = Under (45) 196, over (45) 9 C = Under (45) 194, Over (45) 13 Sex W = Female 32 (15%) C = Female 31 (15%)	W = 208 C = 210 (Consented and completed baseline: W = 201, C = 207)	3-year FU W = 70 C = 63	W = The FitMind program involved three videos featuring senior firefighters talking C = control (training as per usual)	Approximately 30 minutes	Single session
Dimoff and Kelloway (2019) [16]	Canada	Leaders and employees^a^ in two companies (small publishing company and small property management company)	Age leaders: M = 42.58, C = 44. Employees: M = 40.74, C = 38.62. Sex Leaders: M = Female 9 (33.3%), C = Female 4 (30.8%). Employees M = Female 41 (68.3%), C = Female 14 (63.6%)	Leaders M = 40 C = 20	Leaders M = 24 C =13 Employee M = 60 C = 22	M = mental health awareness training; lecture-based modules and interactive case studies and videos + the SOS tool＋a training binder C = Waitlist control	3 hours	Once
Amsalem et al. (2022) [17]	USA	Healthcare workers	Age 34.8, Sex: Female 260 (74%)	VB = 115, V = 114 C = 121	Post VB = 115 V = 114 C = 121 14-day FU VB = 98 V = 97 C = 102 30-day FU VB = 98 V = 97 C = 102 Analyzed VB = 115 V = 114 C = 121	VB = A brief video-based intervention (social contact-based video) + booster video (social contact-based video) V = A single brief video-based intervention (social contact-based video) C = No intervention	3-minute video	VB = A brief video-based intervention on day 1 coupled with a booster video on day 14 V = A single brief video-based intervention
Gärtner et al. (2013) [18]	Netherlands	Nurses and allied healthcare professionals	Age mean age = 42 Sex W = women 156 (82%) C = women 145 (77%)	W = 591, C = 561 (Completed baseline questionnaire W = 191, C = 188)	3 months W = 133 C = 148 6 months W = 115 C = 140 Analysis W = 108 C = 132	W = The mental module for workers’ health surveillance (an online screening and invitation to consult with an occupational physician) C = control	Unclear	Workers’ health surveillance ＝ one time, Consultation with an occupational physician = unclear
Linkins et al. (2011) [19]	USA	Working person with mental illness	Age Over the age of 35 years = 63% Sex Female = 63%	I = 1,257 C = 300	I = 1,157 C = 262	I = SWSW; comprehensive set of support services + navigator + a wellness and employment success plan C = Usual care	Unclear	An initial assessment + occasional contact with a navigator + using as many or as few of the services offered by SWSW (1 year)
Wong et al. (2020) [20]	Singapore	Filipina Foreign Domestic Workers	Age 38.6, Sex: Female only	I = 19 C = 20	I = 18 C = 19	I = CBT-based paraprofessional training (sessions comprised didactics, discussions, and role-plays to enhance learning of support skills to address depression + handouts and homework exercises to consolidate the skills learned ). C = Waitlist group	3 hours per session	Once a week for 4 weeks
Griffiths et al. (2016) [21]	Australia	Employees from a large Australian multi-department government organization	Age 45, Sex: Female = over two-thirds	I = 255 C = 252	Post I = 187 C = 199 FU I = 125 C = 154 Analysis I = 255 C = 252	I = An online psychoeducation workplace induction program (MH-Guru). Two modules focused on depression and generalized anxiety disorder. Interactive format containing graphics and in-program exercises + video vignettes of consumers with lived experience of depression or anxiety, C = a waitlist control	Each module takes approximately 30 minutes to complete	Two modules; one module of MH-Guru per week
Reynolds and Bennett and Lehman (2015) [22]	United States	Employees from small businesses (<500 employees)	Age 18–30: 237 (18%) 31–40: 323 (24%) >40: 780 (58%) Sex Female 746 (56%)	TA = 735 Cho = 372 C = 403	Post TA = 486 Cho = 267 C = 293 FU TA = 416 Cho = 249 C = 203	TA = A shortened version of the Team Awareness Program (information, games, role-playing, and other activities on risks and strengths in the workplace, communication, and peer referral skills) Cho = Choices in health promotion (an adaptive intervention that is customized based on a needs assessment + goal setting + facilitators) C = No training	TA & Cho = 4 hours	TA and Cho = one time
Moll et al. (2018) [23]	Canada	Employees in large hospitals	Age 18 to 69 years; Sex: Female = 170	BS = 97 MHFA = 95	Post BS = 77 MHFA = 90 FU BS = 70 MHFA = 80	BS = Beyond Silence. A contact-based workplace education program co-led by employees who personally experienced mental health issues. MHFA = A standardized mental health literacy training program led by a trained facilitator	BS = 2 hours in-person sessions MHFA = one day	BS = Six 2-hour, in-person sessions, alternating with five online sessions. The in-person sessions were held every other week for a period of 3 months. MHFA = Two full-day sessions were scheduled 1 week apart

Quality Assessment

The quality rating scores based on the Modified Jadad Scale ranged from three to five points (Table 2). One study [14] was considered to be of low quality (three points or less), and the remaining nine [15-23] were considered high quality (four points and above). No study was described as double-blind, and no study included the methods for assessing adverse effects.

**Table 2:** Summary of the results of the selected studies ^a^Number of employees per experimental condition and associated percentages. ^b^The total scores of the follow-up assessment questions were summed and divided by four. ^c^Aggregated responses to the questions, which constitute the total score for "willingness to use resources," with each source of support weighted equally. ^d^The effect sizes within groups, calculated by the authors ^e^11 caregivers (psychologist, psychiatrist, general practitioner, occupational physician, physiotherapist, supervisor, coach, in-company social workers, social workers, religious counselors, and alternative therapeutic counselors). ^f^% represents workers who had consulted one or more caregivers from the group of workers screened as positive at baseline. ^g^Nine categories. ^h^Measured at baseline and at posttest. ^i^Pretraining responses were recorded to indicate whether the participants received help for any reason in the six months before the study. Post-training and follow-up responses were recorded to indicate the receipt or non-receipt of help for any reason during the six months after training. ^j^The participants could endorse more than one of the behaviors, and a summative score of the number of behaviors would be used to measure change in help-seeking. ^k^The investigation was limited to those reporting a mental health experience. C: control; Cho: choices in health promotion; EAP: employee assistance program; FRNSW: Fire and Rescue New South Wales; FU: follow-up; I: informational training; M: mental health awareness training; NS: no significant; OR: odds ratio; SWSW: stay well, stay working; T: team training; TA: a shortened version of the team awareness program; V: a single brief video-based intervention; VB: a brief video-based intervention + booster video; W: the mental module for workers’ health surveillance

Study	Outcome Measures	Assessment Times	Results (Post)	Results (Follow-up)	Quality Rating
Bennett and Lehman (2001) [14]	(1) Attitudes ・EAP helpfulness using one item ・EAP confidentiality using one item (2) Behavior ・Sought help or was encouraged to do so in the last 6 months	Pretest 2 to 4 weeks prior to training; Posttest 2 to 4 weeks following training, FU 6 months following posttest	EAP helpfulness ・No significant differences between intervention and control groups. EAP confidentiality ・No significant differences between intervention and control groups.	Sought help or was encouraged to do so^a^ T = pre 3(5%), Follow-up 8(13%), p <.05. I = pre 1 (2%), Follow-up 4(6%), p <.05. C = pre 5(7%), Follow-up 2(3%), NS.	3/8 (Low)
Tan et al. (2021) [15]	(1) Intention (At baseline) ・Likelihood of seeking help from someone using a single question (At follow-up assessment) ・Likelihood of seeking help from (a) mates or friends in FRNSW, (b) internal help from FRNSW, (C) network of outside help, or (d) health professionals^b^	Pretest Prior to the intervention FU 6-month 1-year 2-year 3-year		Likelihood of help-seeking ・No significant interaction of study condition and time.	5/8 (High)
Dimoff and Kelloway (2019) [16]	(1) Intention ・Employee willingness to use resources in the next 6 weeks from (a) their leader, (b) EAP, or (c) another resource offered by the organization; a three-item measure derived from the General Help-Seeking Questionnaire^c^ (2) Behavior ・Employee resource use; asked about whether available resources had been used in the specified 6-week time period	Pretest 1 week before the training FU 6 weeks posttest 12 weeks posttest	-	Employee willingness to use resources ・Significant group differences over time were observed (p < .01). Pre to 6 weeks ・M: Cohen’s d = 0.61^d^ Pre to 12 weeks ・M: Cohen’s d = 0.51^d^ 6 weeks to 12 weeks ・M & C: NS Employee resource use ・Significant group differences over time were observed (p <.01). Pre to 6 weeks ・M: Cohen’s d = 0.67^d^ Pre to 12 weeks ・M: Cohen’s d = 0.87^d^ 6 weeks to 12 weeks ・M & C: NS	4/8 (High)
Amsalem et al. (2022) [17]	(1) Intention ・Treatment-seeking intentions measured using three “openness to help-seeking” items from the Attitudes Towards Seeking Professional Psychological Help Scale	Pretest Before randomizing participants Post Immediately following the intervention 14 and 30 days following the initial intervention	Treatment-seeking intentions pre to post ・Group × time interaction (p < .001) ・VB: Cohen’s d = 0.50. ・V: Cohen’s d = 0.46. Post to 14 day FU ・Group × time interaction (p < .05) ・VB: Cohen’s d = 0.24 Pre to 30 day FU ・Group × time interaction (p < .001) ・VB: Cohen’s d = 0.74 ・V: Cohen’s d = 0.33 14 day FU to 30 day FU ・Group × time interaction (NS)		4/8 (High)
Gärtner et al. (2013) [18]	(1) Intention ・Intention to seek formal help (yes/no) (2) Behavior ・Help-seeking behavior (formal help): visiting at least 1 of the 11 caregivers^e^ ・Informal help-seeking behavior toward family or friends (yes/no)	Pretest Baseline FU 3 months after baseline 6 months after baseline		Intention to seek formal help ・Group × time interaction (NS) Help-seeking behavior (formal help)^f^ ・Group × time interaction (p = .016) W = baseline 65%, 3-mon 67%, 6-mon 48% C = baseline 63%, 3-mon 54%, 6-mon 56% Informal help-seeking behavior^f^ ・Group × time interaction (NS)	5/8 (High)
Linkins et al. (2011) [19]	(1) Behavior ・Healthcare utilization^g^	Pretest Baseline FU 12 months post-enrollment	Healthcare utilization Baseline SWSW = 804 (69.5%), C = 176 (67.2%), NS.	Healthcare utilization 12 months SWSW = 1141 (98.6%), C = 128 (48.9%), p ≤ .001.	5/8 (High)
Wong et al. (2020) [20]	(1) Attitudes ・Attitudes toward seeking professional help for psychological problems (Attitudes Toward Seeking Professional Psychological Help Scale–Short Form)	Pretest Baseline Posttest Post-training	Attitudes toward seeking professional help for psychological problems Baseline to Post ・The groups did not differ significantly on changes from pre- to post-training	-	5/8 (High)
Griffiths et al. (2016) [21]	(1) Attitudes ・Attitudes to seeking psychological help (Attitudes Toward Seeking Professional Psychological Help Scale–Short Form) (2) Intention ・Help-seeking intentions for depression and anxiety (Separate versions of the Generalized Help-Seeking Questionnaire) (3) Behavior ・Self-reported help-seeking ^h^ (Single question: "In the last two weeks, have you sought information or treatment for depression or anxiety")	Pretest 1 week before intervention Posttest 1 week after completion of the intervention FU 6 months	Attitudes toward seeking psychological help ・No statistically significant interaction between group and time. Help-seeking intentions for depression and anxiety ・No statistically significant interaction between group and time. Self-reported help-seeking ・A statistically significant interaction between group and time was observed for self-reported information/treatment seeking for depression, with help-seeking greater among the intervention group (OR = 1.84, p = .024). ・A significant difference in help-seeking was observed for anxiety in favor of the intervention (OR = 1.90, p = .026).	-	5/8 (High)
Reynolds and Bennett (2015) [22]	(1) Attitudes Help-seeking attitude (willingness to seek help). ・Three items asked about willingness to get help for depression, stress, or a drug or alcohol problem. The mean of these three items was used Attitude to seek help for personal problems (Willing to seek help for a personal problem) ・Depression and stress items were combined. Both by using the mean. Willingness to get help for alcohol or drugs (Willingness to seek help for drug or alcohol problems) ・Single item for alcohol or drugs (2) Behavior Received counseling ・Two items stated, "Please indicate whether you have called or talked to a counselor, mental health professional, EAP, or spiritual or religious counselor for help with…." either a personal problem or a drug or alcohol problem^i^	Pretest 1 month before Posttest 1 month later or 2 weeks following training FU 6 months following posttest	Attitudes Willingness to seek help ・A significant interaction effect of training condition and time was observed (p = .013). ・Employees in TA condition increased their attitudes significantly from pretest to posttest relative to C condition (p = .009), and this relative increase persisted through the 6-month follow-up period (p = .04). ・Employees in Cho condition increased their attitudes significantly from pretest to posttest relative to C condition (p = .04), but their willingness to seek help waned to pretest levels after 6 months (NS). Willingness to seek help for a personal problem ・A significant interaction time-by-condition effect was observed (p = .002). Pre to Post ・TA = pre 2.78 to post 3.20 (p < .05) ・Cho = pre 2.49, to post 2.89 (p < .05) ・C = NS Willing to seek help for drug or alcohol problems ・No time × condition effect	Attitudes Willingness to seek help ・See results (post) Willingness to seek help for a personal problem Pre to FU ・TA = pre 2.78 to FU 3.18 (p < .05). ・Cho = pre 2.49 to FU 2.71 (NS). ・C = NS Received counseling Any reason (either personal problem or a drug or alcohol problem) ・Significant interaction time-by-condition effect observed (p = .03). ・TA = pre 0.08 to post to FU 0.20 (p < .05) ・Cho = pre 0.09 to post to FU 0.15 (NS) ・C = pre 0.05 to post to FU 0.21 (p < .05)	4/8 (High)
Moll et al. (2018) [23]	(1) Attitudes ・The standardized Attitudes Towards Seeking Professional Psychological Help Scale (2) behavior. ・Participants were asked to report whether they accessed any services from a list of 10 health, workplace, and community service options^j ^[24]	Pretest Baseline Posttest Immediately following program completion FU 3 months following program completion	Attitudes^k^ No interactions for treatment arm by time. Help-seeking behavior^k^ ・No evidence of a treatment arm by time interaction either at posttest or follow-up.		5/8 (High)

Year of Publication and Study Location

The years of publication ranged from 2001 to 2022. The countries in which the studies were conducted included the United States (four studies) [14,17,19,22], Canada (two studies) [16,23], Australia (two studies) [15,21], the Netherlands (one study) [18], and Singapore (one study) [20].

Participants

The number of participants ranged from 39 to 1,557. In terms of their occupation, three participants were healthcare workers or employees in large hospitals [17,18,23], two were company employees or leaders [16,22], two were public officers [14,21], one was a firefighter [15], one was a working person with mental illness [19], and one was a Filipina foreign domestic worker [20]. With regard to their sex, nine articles [14-19,21-23] included both male and female participants, and one article [20] included only female participants.

Contents of Interventions

The contents of the interventions included team training and informational training [14], the FitMind program [15], mental health awareness training [16], a single brief video-based intervention and the same brief video intervention plus booster [17], the mental module for workers' health surveillance [18], "Stay Well, Stay Working" [19], cognitive behavioral therapy (CBT)-based paraprofessional training [20], Mental Health Guru [21], a shortened version of the Team Awareness Program and Choices in Health promotion [22], and Beyond Silence [23] (Table 1).

Length of Interventions

The duration of the interventions ranged from three minutes to four hours and from one to 11 sessions. The maximum length of the interventions was one year.

Comparisons

The control type included one active control [23] and nine inactive controls [14-22]. The content of active control includes Mental Health First Aid [23].

Outcome Measures

Five studies targeted help-seeking attitudes [14,20-23], five targeted help-seeking intention [15-18,21], and seven targeted help-seeking behavior [14,16,18,19,21-23]. Only one study targeted these three help-seeking aspects [21]. To measure help-seeking attitudes, three studies used the Attitudes Toward Seeking Professional Psychological Help Scale-Short Form (ATSPPH-SF) [20,21,23]. All sources of help-seeking attitudes were formal.

Regarding the measurement of help-seeking intention, two studies used the General Help-Seeking Questionnaire [16,21], and one study adopted three items from the ATSPPH-SF [17]. The sources of help-seeking intention included three formal sources [16-18], a combination of formal and informal sources [15], and one unclear source [21].

For help-seeking behavior, the studies posed the question regarding whether participants sought help or used resources. The sources of help-seeking behavior included five formal [16,18,19,22,23], one informal [18], and two unclear sources [14,21].

Findings: Help-Seeking Attitudes

Only two out of seven interventions [22] to measure help-seeking attitudes discovered effects at posttest. The remaining five interventions [14,20,21,23] reported no effect. Only one intervention [23] that measured help-seeking attitudes at follow-up (>one month to <six months) reported no effect. Only one of three interventions [22] to measure help-seeking attitudes at follow-up (≥six months to <one year) revealed an effect; the remaining two interventions [21,22] produced no effect. However, Reynolds and Bennett [22] did not find any effect for the willingness to seek help for drug or alcohol problems.

Findings: Help-Seeking Intention

Two of three interventions [17] to measure help-seeking intention found an effect at posttest, whereas one [21] found no effect. The effect sizes ranged from 0.33 to 0.74 [17]. One of two interventions [16] to measure help-seeking intention at follow-up (>one month to <six months) produced an effect, but one intervention [18] did not. The effect sizes ranged from 0.51 to 0.61. The three interventions [15,18,21] at follow-up (≥six months to <one year) produced no effect. Only one intervention [15] at follow-up (one, two, and three years) found no effect.

Findings: Help-Seeking Behavior

One of the two interventions [21] to measure help-seeking behavior produced effects at posttest, whereas the other one [23] produced no effect. For the intervention with effects, the odds ratios of help-seeking behavior for depression and anxiety were 1.84 and 1.90, respectively [21].

Two of three interventions [16,18] to measure help-seeking behavior at follow-up (>one month to <six months) reported effects, whereas the other intervention [23] exhibited no effect. The effect sizes ranged from 0.67 to 0.87. Three out of five interventions [14,22] at follow-up (≥six months to <one year) produced effects, whereas the remaining two interventions [18,22] produced no effects. Only one intervention [19] at the one-year follow-up produced effects.

Findings: Informal Help

Only one intervention [18] to measure informal help exhibited no effect at the three- and six-month follow-ups.

Discussion

This systematic review failed to draw conclusions about the effects of interventions to promote help-seeking related to mental health for workers due to the lack of intervention studies. However, it summarized the effects of these interventions and identified the current situations and challenges of intervention research for workers.

Nearly all interventions to measure help-seeking attitude at posttest and follow-up exhibited no effect. A previous systematic review, which was not limited to the participants, discovered an effect on help-seeking attitude [8]. The results suggest that improving the help-seeking attitudes of workers through interventions is difficult. Additionally, a previous study pointed out that avoiding the disclosure of mental health problems was a default position of workers because of the fear of being stigmatized in the workplace [25]. Thus, the help-seeking attitudes of workers toward mental health may be difficult to improve because of their strong fear that having mental health problems may adversely influence their promotion in the workplace or, worse, lead to unfair retrenchment. Therefore, positive perceptions of not only workers but also colleagues and managers should be encouraged to improve help-seeking attitudes related to mental health for workers.

Only three interventions measured help-seeking intentions at posttest. Two of them reported a wide range of effects at posttest. These findings indicate that short-term effects on help-seeking intention can be expected. Although a previous meta-analysis mentioned the effect on formal help-seeking intention, the effect size was small [9], which is consistent with the findings from the current study. However, in the present systematic review, none of the interventions exhibited any effect at follow-up beyond six months. In other words, no long-term effects were observed. A previous systematic review revealed that one study exhibited big effect sizes at the six-month follow-up [9]. These studies indicate the need to elucidate long-term effects on help-seeking intention.

Only one of two interventions measuring help-seeking behavior at posttest exhibited effects. Approximately half of the interventions were ineffective at follow-ups of less than one year. A previous meta-analysis, which was not limited to the participants, demonstrated an effect on formal help-seeking behavior at posttest and follow-up [9]. The results indicate that promoting help-seeking behavior about mental health for workers is difficult. Another study reported that the norms of the organizations to which workers belonged influenced help-seeking [26]. Thus, workplace climate strongly influences the help-seeking behaviors of workers, potentially rendering the effects of interventions difficult to detect. However, because only one intervention revealed effects at one-year follow-up, a longer follow-up period will be required to determine the effect of help-seeking behavior. Actual help-seeking behavior occurs after an individual recognizes a mental health disorder and understands the need for and access to help. In other words, help-seeking behavior may take a long time. Thus, long-term follow-up is required.

Only one intervention that measures informal help revealed no effect. A previous systematic review, which was not limited to the participants, highlighted the need to conduct intervention studies that target informal help-seeking [9]. Another study illustrated the relationship between social capital and mental health among workers in the workplace [27]. Thus, intervention studies to promote informal help-seeking for workers are necessary.

This systematic review identified a few RCT-related articles for inclusion. However, further RCTs are needed to synthesize the results of each study using statistical analyses. In terms of quality assessment, only one out of 10 studies was of low quality. However, no study was described as double-blind, and no study included methods for assessing adverse effects. Of the selected studies, only one described an active control. Therefore, further double-blind studies should be conducted using an active control. Moreover, intervention studies that target mental health should include a description of the methods used to assess adverse effects and results because of the potential negative influence of the varying mental health conditions of participants.

Another noteworthy aspect is that the selected studies were conducted mainly in North America or Australia, highlighting the need for studies conducted in Asia and Africa, because occupational health systems differ across continents and countries. A previous study indicates that help-seeking related to mental health was unsuccessful in developing countries [5], suggesting the need for studies to be conducted in such countries.

Moreover, participants' occupations were wide-ranging and included company employees, public officers, and healthcare workers. Because workplace climate, including attitudes toward mental health, differs according to the type of occupation, further research is essential to analyze such needs according to the type of occupation.

Both the duration and content of interventions varied widely, underscoring the need to accumulate studies to determine effective content and durations for interventions. The present study also identified diverse outcome measures of help-seeking without using standard measures, which are needed to compare effects across studies. Furthermore, the help-seeking attitudes related to the mental health of workers, which have varying impacts, such as on workplace promotions, may differ from those of other groups. A previous study mentioned that help-seeking among workers was related to its perceived influence on their careers [28]. Thus, developing a scale that can appropriately measure help-seeking attitudes related to mental health for workers may be necessary.

## Conclusions

Although this systematic review was unable to draw conclusions about the effects of interventions on help-seeking related to the mental health of workers due to the lack of intervention studies, it identified the effects and challenges of interventions for workers. The results highlight the difficulty in improving help-seeking related to mental health for workers compared to other groups. Therefore, further research is required to enhance help-seeking tailored to the complex situations faced by workers.

## Appendices

**Table 3:** Search strategy of five databases (The Cochrane Library, PubMed, CINAHL via EBSCOhost, PsycINFO via EBSCOhost, Ichushi-Web)

#	Query (PubMed; Final Search: February 1, 2022)	Results
45	(((((((((((((((help-seeking behavior[MeSH Terms]) OR ((seek*[Title/Abstract]) AND (help[Title/Abstract]))) OR ((seek*[Title/Abstract]) AND (treatment[Title/Abstract]))) OR ((seek*[Title/Abstract]) AND (care[Title/Abstract]))) OR ((seek*[Title/Abstract]) AND (service[Title/Abstract]))) OR (service use[Title/Abstract])) OR (service utilization[Title/Abstract])) OR (health care utilization[Title/Abstract])) OR (help-seeking attitudes[Title/Abstract])) OR (help-seeking intentions[Title/Abstract])) OR (help-seeking behaviors[Title/Abstract])) OR (help-seeking behavior[Title/Abstract])) OR (help-seeking behaviour[Title/Abstract])) OR (help-seeking behaviours[Title/Abstract])) AND (((((((((((((mental health[MeSH Terms]) OR (mental health[Title/Abstract])) OR (mental illness[Title/Abstract])) OR (mental illnesses[Title/Abstract])) OR (mental disorder[Title/Abstract])) OR (mental disorders[Title/Abstract])) OR (mental disorders[MeSH Terms])) OR (mental health problem[Title/Abstract])) OR (mental health problems[Title/Abstract])) OR (depression[Title/Abstract])) OR (depression[MeSH Terms])) OR (anxiety[Title/Abstract])) OR (anxiety[MeSH Terms]))) AND (((((((((((worker) OR (employee)) OR (workforce)) OR (labor)) OR (labor force)) OR (staff)) OR (manager)) OR (owner)) OR (workplace)) OR (worksite)) OR (occupational health))	1,029
44	(((((((((((((((help-seeking behavior[MeSH Terms]) OR ((seek*[Title/Abstract]) AND (help[Title/Abstract]))) OR ((seek*[Title/Abstract]) AND (treatment[Title/Abstract]))) OR ((seek*[Title/Abstract]) AND (care[Title/Abstract]))) OR ((seek*[Title/Abstract]) AND (service[Title/Abstract]))) OR (service use[Title/Abstract])) OR (service utilization[Title/Abstract])) OR (health care utilization[Title/Abstract])) OR (help-seeking attitudes[Title/Abstract])) OR (help-seeking intentions[Title/Abstract])) OR (help-seeking behaviors[Title/Abstract])) OR (help-seeking behavior[Title/Abstract])) OR (help-seeking behaviour[Title/Abstract])) OR (help-seeking behaviours[Title/Abstract])) AND (((((((((((((mental health[MeSH Terms]) OR (mental health[Title/Abstract])) OR (mental illness[Title/Abstract])) OR (mental illnesses[Title/Abstract])) OR (mental disorder[Title/Abstract])) OR (mental disorders[Title/Abstract])) OR (mental disorders[MeSH Terms])) OR (mental health problem[Title/Abstract])) OR (mental health problems[Title/Abstract])) OR (depression[Title/Abstract])) OR (depression[MeSH Terms])) OR (anxiety[Title/Abstract])) OR (anxiety[MeSH Terms]))) AND (((((((((((worker) OR (employee)) OR (workforce)) OR (labor)) OR (labor force)) OR (staff)) OR (manager)) OR (owner)) OR (workplace)) OR (worksite)) OR (occupational health))	1,028
43	(((((((((((((((help-seeking behavior[MeSH Terms]) OR ((seek*[Title/Abstract]) AND (help[Title/Abstract]))) OR ((seek*[Title/Abstract]) AND (treatment[Title/Abstract]))) OR ((seek*[Title/Abstract]) AND (care[Title/Abstract]))) OR ((seek*[Title/Abstract]) AND (service[Title/Abstract]))) OR (service use[Title/Abstract])) OR (service utilization[Title/Abstract])) OR (health care utilization[Title/Abstract])) OR (help-seeking attitudes[Title/Abstract])) OR (help-seeking intentions[Title/Abstract])) OR (help-seeking behaviors[Title/Abstract])) OR (help-seeking behavior[Title/Abstract])) OR (help-seeking behaviour[Title/Abstract])) OR (help-seeking behaviours[Title/Abstract])) AND (((((((((((((mental health[MeSH Terms]) OR (mental health[Title/Abstract])) OR (mental illness[Title/Abstract])) OR (mental illnesses[Title/Abstract])) OR (mental disorder[Title/Abstract])) OR (mental disorders[Title/Abstract])) OR (mental disorders[MeSH Terms])) OR (mental health problem[Title/Abstract])) OR (mental health problems[Title/Abstract])) OR (depression[Title/Abstract])) OR (depression[MeSH Terms])) OR (anxiety[Title/Abstract])) OR (anxiety[MeSH Terms]))) AND (((((((((((worker) OR (employee)) OR (workforce)) OR (labor)) OR (labor force)) OR (staff)) OR (manager)) OR (owner)) OR (workplace)) OR (worksite)) OR (occupational health))	1,038
42	(((((((((((((((help-seeking behavior[MeSH Terms]) OR ((seek*[Title/Abstract]) AND (help[Title/Abstract]))) OR ((seek*[Title/Abstract]) AND (treatment[Title/Abstract]))) OR ((seek*[Title/Abstract]) AND (care[Title/Abstract]))) OR ((seek*[Title/Abstract]) AND (service[Title/Abstract]))) OR (service use[Title/Abstract])) OR (service utilization[Title/Abstract])) OR (health care utilization[Title/Abstract])) OR (help-seeking attitudes[Title/Abstract])) OR (help-seeking intentions[Title/Abstract])) OR (help-seeking behaviors[Title/Abstract])) OR (help-seeking behavior[Title/Abstract])) OR (help-seeking behaviour[Title/Abstract])) OR (help-seeking behaviours[Title/Abstract])) AND (((((((((((((mental health[MeSH Terms]) OR (mental health[Title/Abstract])) OR (mental illness[Title/Abstract])) OR (mental illnesses[Title/Abstract])) OR (mental disorder[Title/Abstract])) OR (mental disorders[Title/Abstract])) OR (mental disorders[MeSH Terms])) OR (mental health problem[Title/Abstract])) OR (mental health problems[Title/Abstract])) OR (depression[Title/Abstract])) OR (depression[MeSH Terms])) OR (anxiety[Title/Abstract])) OR (anxiety[MeSH Terms]))) AND (((((((((((worker) OR (employee)) OR (workforce)) OR (labor)) OR (labor force)) OR (staff)) OR (manager)) OR (owner)) OR (workplace)) OR (worksite)) OR (occupational health))	14,975
41	((((((((((worker) OR (employee)) OR (workforce)) OR (labor)) OR (labor force)) OR (staff)) OR (manager)) OR (owner)) OR (workplace)) OR (worksite)) OR (occupational health)	5,409,536
40	occupational health	227,266
39	worksite	60,978
38	workplace	62,649
37	owner	20,511
36	manager	3,417,293
35	staff	284,024
34	labor force	151,429
33	labor	1,608,662
32	workforce	103,947
31	employee	807,761
30	worker	876,054
29	((((((((((((mental health[MeSH Terms]) OR (mental health[Title/Abstract])) OR (mental illness[Title/Abstract])) OR (mental illnesses[Title/Abstract])) OR (mental disorder[Title/Abstract])) OR (mental disorders[Title/Abstract])) OR (mental disorders[MeSH Terms])) OR (mental health problem[Title/Abstract])) OR (mental health problems[Title/Abstract])) OR (depression[Title/Abstract])) OR (depression[MeSH Terms])) OR (anxiety[Title/Abstract])) OR (anxiety[MeSH Terms])	1,799,290
28	anxiety[MeSH Terms]	99,710
27	anxiety[Title/Abstract]	227,703
26	depression[MeSH Terms]	239,419
25	depression[Title/Abstract]	383,222
24	mental health problems[Title/Abstract]	15,406
23	mental health problem[Title/Abstract]	1,351
22	mental disorders[MeSH Terms]	1,347,734
21	mental disorders[Title/Abstract]	46,229
20	mental disorder[Title/Abstract]	10,308
19	mental illnesses[Title/Abstract]	5,185
18	mental illness[Title/Abstract]	33,020
17	mental health[Title/Abstract]	187,865
16	mental health[MeSH Terms]	50,358
15	(((((((((((((help-seeking behavior[MeSH Terms]) OR ((seek*[Title/Abstract]) AND (help[Title/Abstract]))) OR ((seek*[Title/Abstract]) AND (treatment[Title/Abstract]))) OR ((seek*[Title/Abstract]) AND (care[Title/Abstract]))) OR ((seek*[Title/Abstract]) AND (service[Title/Abstract]))) OR (service use[Title/Abstract])) OR (service utilization[Title/Abstract])) OR (health care utilization[Title/Abstract])) OR (help-seeking attitudes[Title/Abstract])) OR (help-seeking intentions[Title/Abstract])) OR (help-seeking behaviors[Title/Abstract])) OR (help-seeking behavior[Title/Abstract])) OR (help-seeking behaviour[Title/Abstract])) OR (help-seeking behaviours[Title/Abstract])	111,308
14	help-seeking behaviours[Title/Abstract]	253
13	help-seeking behaviour[Title/Abstract]	745
12	help-seeking behavior[Title/Abstract]	957
11	help-seeking behaviors[Title/Abstract]	550
10	help-seeking intentions[Title/Abstract]	239
9	help-seeking attitudes[Title/Abstract]	183
8	health care utilization[Title/Abstract]	8,401
7	service utilization[Title/Abstract]	5,775
6	service use[Title/Abstract]	7,551
5	(seek*[Title/Abstract]) AND (service[Title/Abstract])	11,082
4	(seek*[Title/Abstract]) AND (care[Title/Abstract])	47,631
3	(seek*[Title/Abstract]) AND (treatment[Title/Abstract])	46,589
2	(seek*[Title/Abstract]) AND (help[Title/Abstract])	23,152
1	help-seeking behavior[MeSH Terms]	1,076

#	Query (CINAHL via EBSCOhost; Final Search: February 1, 2022)	Results
S1	TI help seeking behavior OR AB help seeking behavior	1,483
S2	TI service use OR AB service use	10,248
S3	TI service utilization OR AB service utilization	6,087
S4	TI health care utilization OR AB health care utilization	11,377
S5	TI help seeking attitudes OR AB help seeking attitudes	221
S6	TI help seeking intentions OR AB help seeking intentions	221
S7	MH help seeking behavior	7,535
S8	TI seek* AND (TI help OR TI treatment OR TI care OR TI service)	6,539
S9	AB seek* AND (AB help OR AB treatment OR AB care OR AB service)	47,512
S10	S1 OR S2 OR S3 OR S4 OR S5 OR S6 OR S7 OR S8 OR S9	77,229
S11	MH mental health	47,164
S12	TI mental health OR AB mental health	127,743
S13	TI mental illness OR AB mental illness	22,965
S14	MH mental disorders	63,743
S15	TI mental disorder OR AB mental disorder	20,922
S16	TI mental health problem OR AB mental health problem	12,683
S17	MH depression	119,934
S18	TI depression OR AB depression	138,325
S19	MH anxiety	51,149
S20	TI anxiety OR AB anxiety	92,768
S21	S11 OR S12 OR S13 OR S14 OR S15 OR S16 OR S17 OR S18 OR S19 OR S20	395,310
S22	worker	112,431
S23	employee	59,249
S24	workforce	44,065
S25	labor	60,777
S26	labor force	6,598
S27	staff	174,779
S28	manager	47,288
S29	owner	3,996
S30	workplace	46,307
S31	worksite	18,164
S32	occupational health	59,957
S33	S22 OR S23 OR S24 OR S25 OR S26 OR S27 OR S28 OR S29 OR S30 OR S31 OR S32	493,908
S34	MH randomized controlled trials	126,049
S35	TI randomized controlled trial OR AB randomized controlled trial	101,957
S36	TI randomized clinical trial OR AB randomized clinical trial	33,836
S37	TI randomised controlled trial OR AB randomised controlled trial	30,159
S38	TI rct OR AB rct	25,985
S39	TI randomi* OR AB randomi*	285,725
S40	S34 OR S35 OR S36 OR S37 OR S38 OR S39	323,913
S41	S10 AND S21 AND S33 AND S40	165
S42	S41	0
S43	S41	165
S44	S42 OR S43	165

#	Query (PsycINFO via EBSCOhost; Final Search: February 1, 2022)	Results
S1	DE help seeking behavior	6,201
S2	TI help seeking behavior OR AB help seeking behavior	3,026
S3	TI service use OR AB service use	10,054
S4	TI service utilization OR AB service utilization	5,510
S5	TI health care utilization OR AB health care utilization	5,468
S6	TI help seeking attitudes OR AB help seeking attitudes	737
S7	TI help seeking intentions OR AB help seeking intentions	458
S8	TI seek* AND (TI help OR TI treatment OR TI care OR TI service)	7,378
S9	AB seek* AND (AB help OR AB treatment OR AB care OR AB service)	63,561
S10	S1 OR S2 OR S3 OR S4 OR S5 OR S6 OR S7 OR S8 OR S9	83,184
S11	DE mental health	84,188
S12	TI mental health OR AB mental health	205,840
S13	TI mental illness OR AB mental illness	46,223
S14	DE mental disorders	137,974
S15	TI mental disorder OR AB mental disorder	62,407
S16	TI mental health problem OR AB mental health problem	19,951
S17	TI depression OR AB depression	263,111
S18	DE anxiety	88,568
S19	TI anxiety OR AB anxiety	213,038
S20	DE depression	50,143
S21	S11 OR S12 OR S13 OR S14 OR S15 OR S16 OR S17 OR S18 OR S19 OR S20	703,224
S22	worker	114,848
S23	employee	103,201
S24	workforce	30,770
S25	labor	44,884
S26	labor force	3,954
S27	staff	104,833
S28	manager	61,683
S29	owner	7,617
S30	workplace	60,117
S31	worksite	20,654
S32	occupational health	22,390
S33	S22 OR S23 OR S24 OR S25 OR S26 OR S27 OR S28 OR S29 OR S30 OR S31 OR S32	404,086
S34	DE randomized controlled trials	809
S35	DE randomized clinical trials	301
S36	TI randomized controlled trial OR AB randomized controlled trial	36,520
S37	TI randomized clinical trial OR AB randomized clinical trial	9,570
S38	TI randomised controlled trial OR AB randomised controlled trial	6,953
S39	TI rct OR AB rct	9,156
S40	TI randomi* OR AB randomi*	97,456
S41	S34 OR S35 OR S36 OR S37 OR S38 OR S39 OR S40	99,057
S42	S10 AND S21 AND S33 AND S41	116
S43	S42	0
S44	S42	115
S45	S43 OR S44	115

ID	Search (The Cochrane Library; Final Search: February 1, 2022)	Hits
#1	[mh "help seeking behavior"]	46
#2	("help seeking behavior"):ti,ab	157
#3	("seek*"):ti,ab	4640
#4	("help"):ti,ab	45173
#5	("treatment"):ti,ab	736126
#6	("care"):ti,ab	190123
#7	("service"):ti,ab	21669
#8	#3 AND (#4 OR #5 OR #6 OR #7)	3667
#9	("service use"):ti,ab	1188
#10	("service utilization"):ti,ab	995
#11	("health care utilization"):ti,ab	1552
#12	("help seeking attitudes"):ti,ab	46
#13	("help seeking intentions"):ti,ab	72
#14	("help seeking behaviors"):ti,ab	69
#15	("help seeking behaviour"):ti,ab	157
#16	("help seeking behaviours"):ti,ab	69
#17	#1 OR #2 OR #8 OR #9 OR #10 OR #11 OR #12 OR #13 OR #14 OR #15 OR #16	7425
#18	[mh "mental health"]	1820
#19	("mental health"):ti,ab	19523
#20	("mental illness"):ti,ab	4033
#21	("mental illnesses"):ti,ab	635
#22	[mh "mental disorders"]	79057
#23	("mental disorder"):ti,ab	1146
#24	("mental disorders"):ti,ab	4745
#25	("mental health problem"):ti,ab	176
#26	("mental health problems"):ti,ab	1545
#27	[mh "depression"]	13537
#28	("depression"):ti,ab	72754
#29	[mh "anxiety"]	8779
#30	("anxiety"):ti,ab	51471
#31	#18 OR #19 OR #20 OR #21 OR #22 OR #23 OR #24 OR #25 OR #26 OR #27 OR #28 OR #29 OR #30	169257
#32	worker	5068
#33	employee	2216
#34	workforce	1186
#35	labor	17289
#36	labor force	228
#37	staff	23536
#38	manager	8671
#39	owner	237
#40	workplace	3445
#41	worksite	902
#42	occupational health	7973
#43	#32 OR #33 OR #34 OR #35 OR #36 OR #37 OR #38 OR #39 OR #40 OR #41 OR #42	59764
#44	#17 AND #31 AND #43	580
#45	[mh "randomized controlled trial"]	119
#46	("randomized controlled trial"):ti,ab	181958
#47	("randomized controlled trials"):ti,ab	27718
#48	("randomized clinical trial"):ti,ab	52832
#49	("randomized clinical trials"):ti,ab	7953
#50	("randomised controlled trial"):ti,ab	181981
#51	("randomised controlled trials"):ti,ab	27713
#52	("rct"):ti,ab	32216
#53	("rcts"):ti,ab	11591
#54	("randomi*"):ti,ab	34
#55	#45 OR #46 OR #47 OR #48 OR #49 OR #50 OR #51 OR #52 OR #53 OR #54	272608
#56	#44 AND #55	305

Ichushi-Web (Final Search: January 27, 2022)	Results
(((((((援助要請行動/TH) or (援助要請行動/TA) or (援助探求行動/TA) or (求助行動/TA) or (相談行動/TA) or (被援助行動/TA) or (被援助志向性/TA) or (サービス利用/TA)) or (受援/TA)) or (受診行動/TA)) or (援助要請/TA) or (援助要請意図/TA) or (援助要請態度/TA)) and ((精神保健/TH) or (精神保健/TA) or (精神衛生/TA) or (メンタルヘルス/TA) or (心の健康/TA) or (こころの健康/TA) or (精神健康/TA) or (精神的健康/TA) or (精神疾患/TH) or (精神疾患/TA) or (精神病/TH) or (精神病/TA) or (抑うつ/TH) or (抑うつ/TA) or (うつ/TA) or (不安/TH) or (不安/TA)) and (((労働者/TH or 労働者/AL)) or ((労働者/TH or 勤労者/AL)) or ((労働者/TH or 就労者/AL)) or (従業員/AL) or ((労働力/TH or 労働力/AL)) or ((労働/TH or 労働/AL)) or (職員/AL) or (スタッフ/AL) or ((管理者/TH or 管理者/AL)) or ((管理者/TH or 管理職/AL)) or (経営者/AL) or ((職場/TH or 職場/AL)) or (事業場/AL) or ((労働衛生/TH or 産業保健/AL))))) and (LA=日本語,英語 and RD=ランダム化比較試験)	0
